# Promoter Cre‐Specific Genotyping Assays for Authentication of Cre‐Driver Mouse Lines

**DOI:** 10.1002/jbm4.10128

**Published:** 2019-01-18

**Authors:** Greig Couasnay, Christopher Frey, Florent Elefteriou

**Affiliations:** ^1^ Department of Orthopedic Surgery; ^2^ Departments of Human and Molecular Genetics Baylor College of Medicine Houston TX USA

**Keywords:** CRE‐RECOMBINASE, TRANSGENIC MICE, FLOXED ALLELE, MOUSE MODEL, SPECIFICITY, PCR, GENOTYPING

## Abstract

The Cre‐LoxP system gene knockout (KO) technology provides cell‐ and time‐specificity of gene ablation to investigate cell‐autonomous gene function *in vivo*, and is paramount for understanding the function of genes involved in bone development, remodeling, and repair. This approach permits gene ablation in a cell‐ or tissue‐specific, differentiation stage‐specific, and inducible manner, thanks to the use of well‐chosen promoters that drive expression of the Cre recombinase in selected cells/tissues. The generation of these powerful tools has led to the expansion of Cre mouse lines available to the research community, which are often shared within and between laboratories. Although convenient and commonly used, genotyping these Cre lines with a generic set of primers that amplifies the *Cre* transgene does not distinguish between various Cre‐deleter lines. This practice poses the significant risk of mistakenly swapping Cre lineages, as laboratories often host and handle several lines at a time and utilize multiple lines per project. In line with the NIH‐led effort to promote authentication of biological reagents and increase scientific rigor, we report here strategies for designing appropriate sets of primers able to discriminate some of most widely used Cre‐deleter mouse lines in the field of bone biology, and the validation of 24 of them. © 2018 The Authors *JBMR Plus* published by Wiley Periodicals, Inc. on behalf of American Society for Bone and Mineral Research.

## Introduction

The Cre‐LoxP system gene knockout (KO) technology uses the bacteriophage P1 Cre recombinase (Cre) to catalyze the excision of selected DNA sequences flanked by LoxP sequences.^1^, ^2^, ^3^, ^4^, ^5^ Cre is a 38 kDa integrase that catalyzes recombination between LoxP sites, which are 34‐bp consensus sequences consisting of an 8‐bp core‐spacer sequence flanked by an inverted 13‐bp repeat.^6^ Judicious insertion of these LoxP sequences within the genome allows for excision of defined sequences resulting in elimination, modification, or activation of gene function. Orientation of LoxP sites can be manipulated to either delete, invert, duplicate, or translocate part of chromosomes as well.^7^, ^8^ The prokaryotic coding sequence of the Cre recombinase is most commonly used, although more recently, it was modified (called “iCre”) by applying mammalian codons to decrease the high CpG content of the original sequence, thus reducing the likelihood of epigenetic mammalian silencing.^9^, ^10^

**Figure 1:**
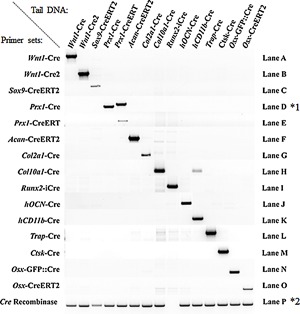
Validation of amplification specificity for each primer sets. All PCRs were performed with the same thermal cycle (see Materials & Methods). (*1) = the primer set for *Prx1‐Cre* also recognizes the *Prx1‐CreERT*, but generates a larger amplicon; (*2) = the Cre‐recombinase PCR is not able to recognize the codon‐improved Cre recombinase used in *Runx2‐iCre* transgenic mouse.

One of the first advantages of the Cre‐LoxP system is its ability to ablate genes in specific cell lineages or tissues, thus bypassing lethal or profound developmental defects experienced with global KO mice. This precise control of gene modification in selected lineages and/or developmental/differentiation stages allows one to design animal models of human diseases, and to study gene function in a specific tissue in the context of the whole organism. Site of target gene inactivation (summarized in ref 11) in conditional KO mice (cKO) is regulated by the expression pattern of the Cre recombinase, which is driven by tissue/cell‐“specific” promoters (Fig. 1 *A*). Additionally, temporal control of gene inactivation can be leveraged to circumvent problems such as embryonic lethality or developmental abnormalities arising from global gene deletion or early developmental Cre‐recombinase activity. This can be achieved by the induction of Cre expression or activity with tetracycline (or doxycycline),^12^, ^13^, ^14^, type I interferon,^15^ or tamoxifen.^6^, ^16^, ^17^ The latter is the most widely used, facilitated by coupling the Cre‐recombinase sequence with the human estrogen‐receptor‐ligand binding sequence (hER‐LBD).^6^ In response to synthetic estrogen antagonists such as tamoxifen, 4‐hydroxytamoxifen (4‐OHT), or ICI 182,780 (ICI),^16^, ^18^ the chimeric Cre‐hER fusion protein is activated and translocates to the nucleus to induce recombination of floxed sequences. Because the original Cre‐hER fusion protein was responsive to endogenous estrogen too (17β‐estradiol or E2), a mutation on glycine 521 in the hER‐LBD was introduced (G521R) to inhibit its sensitivity to endogenous E2, while conserving its sensitivity to 4‐OHT and ICI (Fig. 1 *B*). This chimeric protein, called Cre‐ERT, was further modified to increase its sensitivity by the insertion of three different mutations in the hER‐LBD sequence (namely G400V, M543A, and L544A), leading to the generation of the highly ligand‐dependent Cre‐ERT2 recombinase protein,^19^ which is 10 times as sensitive to 4‐OHT than Cre‐ER (Fig. 1 *C*).^20^ Contemporaneously, another team developed a transgene that couples the Cre‐recombinase sequence with the ligand‐binding domain of the mouse estrogen receptor mutated at position G525R, to generate a Cre‐recombinase nonsensitive to endogenous estrogen, namely Cre‐ER^TM^ (Fig. 1 *D*).^21^, ^22^

The increasing number of Cre lines available to the scientific community substantially refined our ability to investigate gene function. However, the use of generic PCR primers amplifying *Cre* for genotyping these Cre lines does not discriminate one line from the other. This common practice is associated with the risk of misinterpretation of results and a concern for scientific rigor. In this study, we initiated the design and validation of a series of primer pairs able to discriminate the different Cre lines used in the field of bone biology from each other.

## Materials and Methods

### DNA preparation

Investigators with active Cre‐line colonies were approached and requested to share tail DNA collected for genotyping purposes. DNA was shipped at room temperature and stored at −20°C upon receipt. Tail DNA samples were received in various forms, either unpurified, dry‐frozen, already digested either by proteinase K or alkaline lysis, or already purified. No significant variation in PCR efficiency was observed between samples extracted with different methods.

#### Primer selection

The design of optimum primers for our task first required determination of the DNA elements (promoter, enhancer, exons, and introns) used to drive Cre expression in the original Cre mouse line as published. The genomic sequence of these regions was downloaded from the Ensembl website (http://www.ensembl.org/index.html), using the mouse, rat, or human genome, depending on the constructs. The FASTA sequence of Cre‐driving elements was assembled as described in each original article and pasted upstream of the Cre‐ or iCre‐recombinase sequence fused with the hERT/2 or mouse ER‐binding domain using the SerialCloner 2.6.1 DNA software. Specific forward and reverse primers were selected with Primer Blast (https://www.ncbi.nlm.nih.gov/tools/primer‐blast/) from this in silico sequence to generate PCR products ranging from 300 to 2500 bp (see the Results section).

The sequence for the improved codon Cre recombinase (iCre) was derived from the paavCAG‐iCre plasmid (Addgene plasmid 51904) and the bacteriophage P1 Cre‐recombinase sequence was derived from the pCAG‐Cre plasmid (Addgene plasmid 13775). The human ERT and ERT2 sequences were derived from references 16 and 19, respectively. The mouse ERTM sequence was derived from references 21 and 22.

#### PCR amplification

All primers were purchased from Thermo Fisher Scientific (Waltham, MA, USA). PCR reactions were performed with 10 μL of the Green master mix 2× from Promega (M5123; Promega, San Luis Obispo, CA, USA), 250nM of each primer, 400 μM of each dNTP, 4mM MgCl2, and 1μL of tail gDNA. No difference in PCR efficiency was observed when using another Taq polymerase (DreamTaq; Thermo Fisher Scientific cat. #K1081). A common PCR thermal‐cycle amplification program was used for all primer pairs (1 min at 95°C then 15 s at 95°C, 40 s at 55°C, 120 s at 72°C, repeated 35 times, and a last step of 10 min at 72°C). Sequences for each primer set are provided in Tables 1, 2, and 3. High‐melting‐resolution PCR (HRM PCR) was performed using the LightCycler 480 High Resolution Melting Master mix (cat. #04090631001; Roche Diagnostics, Mannheim, Germany) after addition of MgCl2 (0.2 μM final), primers (0.4μM each; Table 4), and purified genomic DNA (approximatively 10 ng). Amplification and melt curve analyses were then performed on a Roche LightCycler 96 instrument under the following parameters: preincubation 95°C 10 min, amplification 95°C 10 s, 60°C 15 s, 72°C 15 s, HRM 95°C 1 min, 40°C 1 min, 65°C 1 s, 97°C with 15 readings per °C.

**Table 1:** List of Specific Primers to Distinguish the Different Bone Cre‐Driver Transgenic Mice

Cre line	PMID	Primer	Sequence (5’‐3’)	Size amplicon (bp)
*Wnt1‐Cre*	9843687	FwCre	TTGGCAGAACGAAAACGCTG	2251
		Rw1	GGGTTCTGTCGGATCAGTCG	
*Wnt1‐Cre2*	23648512	FwCre	TTGGCAGAACGAAAACGCTG	1323
		Rw2	TGTAGGTTAGGGTTGGGTTGC	
*Sox9‐CreERT2*	20806356	FSox9	CACCTTCACTTACATGAACC	2441
		RErt	AAACAGTAGCTTCACTGGGTG	
2.4‐kb *Prx1‐Cre*	12112875	FPrx	GTCATGAAAACACCGTCCAG	1121
		RCre	CATCGACCGGTAATGCAG	
2.4‐kb *Prx1‐CreERT*	19538944	FPrx	GTCATGAAAACACCGTCCAG	2876
		RErt	AAACAGTAGCTTCACTGGGTG	
*Acan‐CreERT2*	19830818	FNeo	AAGAACTCGTCAAGAAGGCGATAGAAGGCG	1534
		RAcan	GATGCAGTTTGGGTGATGCG	
*Col2a1‐Cre*	10686612	FCol2	TTGATCTTTGGATTCTCGCCC	896
		RCre	CATCGACCGGTAATGCAG	
*Col10a1‐Cre*	18692570	FColX	TTTAGAGCATTATTTCAAGGCAGTTTCCA	305
		RColX	AGGCAAATTTTGGTGTACGG	
*Osx1‐GFP::Cre*	16854976	FGfp	GCCAGGCAGGTGCCTGGACAT	407
		ROsx	CTCTTCATGAGGAGGACCCT	
*Osx‐CreERT2*	20708594	FOsx	CAGGAAATTTGGACCCTCTG	1668
		RErt	AAACAGTAGCTTCACTGGGTG	
*Runx2‐iCre*	20519123	FRunx2	CCAGGAAGACTGCAAGAAGG	600
		RiCre	TGGCTTGCAGGTACAGGAG	
*hOCN‐Cre*	12215457	FOCN	CAAATAGCCCTGGCAGATTC	260
		ROCN	TGATACAAGGGACATCTTCC	
*hCD11b‐Cre*	15754380	FCD11b	AGGCAGGCTAAGTCTGTTCA	390
		RCre	CATCGACCGGTAATGCAG	
*Trap‐Cre*	10750559	FTrap	ATGCTAGAGTCGAGACACCC	867
		RCre	CATCGACCGGTAATGCAG	
*Ctsk‐Cre*	15282744	FCtsk	CCATTCGTGTCTGAGGCTTT	992
		RCre	CATCGACCGGTAATGCAG	
*Cre‐Universal*	//	FCre	GAGTGATGAGGTTCGCAAGA	635
		RCre	CTACACCAGAGACGGAAATC	

**Table 2:** List of Specific Primers to Discriminate the Different *Col1a1 Cre*‐Driver Transgenic Mice

	Primer	Sequence primer (5’‐3’)	Size amplicon (bp)
Rat *Col1a1‐Cre* specific	FCol1R	ACAAGGGTGGCAGAATTGCAAA	499
	RCre	CATCGACCGGTAATGCAG	
Mouse *Col1a1‐Cre* specific	FCol1M	ctcagagctgttatttatta	958
	RCre	CATCGACCGGTAATGCAG	
Rat 3.6‐kb *Col1a1‐Cre* specific	F3.6Col1R	GAGACCCCAAGCACATTCTTC	1749
	R3.6Col1R	tccagccccaggtgtaacag	
Mouse 3.2‐kb *Col1a1‐CreER*	FCol1M	ctcagagctgttatttatta	2732
	RmER	GGTGGACCTGATCATGGAGAT	

**Table 3:** List of Specific Primers to Discriminate the Different *Dmp1 Cre*‐Driver Transgenic Mice

	Primer	Sequence (5’‐3’)	Size amplicon (bp)
All *Dmp1* promoter	FDmp	AATATGAAGCCTGCCACAGC	909
	RCre	CATCGACCGGTAATGCAG	1260
Only 10‐kb *Dmp1* promoter	FDmp5’	GCTAGATTTAAGCTCAACTTT	1978
	RDmp1	TTAAGGGTGGACTTAAGGTG	
10‐kb *Dmp1‐CreERT2*	FwCre	TTGGCAGAACGAAAACGCTG	818
	RErt	AAACAGTAGCTTCACTGGGTG	

**Table 4:** hERT2 Primer Sequences for the High Resolution Melting Curve PCR

	Primer	Sequence (5’‐3’)	Size amplicon (bp)
*hERT2‐HRM*	hERT2‐HRM‐For	ACAGCATGAAGTGCAAGAACG	51
	hERT2‐HRM‐Rev	CCTCCAGCAGCAGGTCATAG	

#### PCR specificity validation

PCR amplicon size and sequence were used to validate the identity of amplification products. PCR products were run on EtBr agarose gels, and size was estimated with comparison to a DNA mass ladder (Thermo Fisher Scientific, cat. #10787018). PCR bands were extracted, purified with the QIAquick gel extraction kit (cat. #28704; QIAGEN, Valencia, CA, USA) and sequenced to verify correct gene amplification. The specificity of each primer set was tested against the other available Cre‐transgenic mouse DNA and is presented in Fig.  1. A PCR using generic primers amplifying the Cre recombinase was used as a positive internal PCR control to detect the presence of the Cre transgene.

## Results

**Figure 2:**
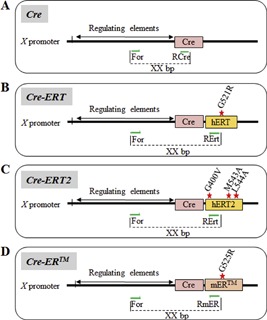
Representation of the different Cre recombinase constructs used to create Cre transgenic mice. A: Generic strategy for primer (green arrow) selection. B: Cre‐ERT construct with the G521R mutation in the ERT moiety of the construct. C: Cre‐ERT2 construct with triple mutations in the ERT moiety of the construct. D: Cre‐ERTM construct with the G525R mutation in the ERT moiety of the construct. Cre: Cre‐recombinase.

We focused this work on Cre lines used to investigate gene function in the bone mesenchymal and monocytic lineages. It was made possible by investigators kindly sharing tail genomic DNA (gDNA) from mice available in their colonies. All tail DNA received were first screened for the presence of the Cre transgene, using generic Cre primers (Cre Universal; Table 1), before testing specific primer sets. Each Cre line for which gDNA was made available to us is listed below, with information regarding: (1) the structure of the promoter elements used to drive Cre‐recombinase expression; (2) the type of Cre recombinase used to distinguish each line from the other listed herein; and (3) the strategy used to generate Cre‐line‐specific amplicons. In most cases, a forward primer sequence was selected in the promoter/enhancer sequence used to drive Cre expression, a reverse primer sequence was chosen in the Cre transgene (Fig. 2 *A*), and the pair of primers was validated by PCR for amplification efficiency and specificity. To distinguish between Cre lines that used the same promoter from different species (*Dmp1* and *Col1a1‐Cre* lines), primers were chosen in rare nonhomologous promoter regions. Global (iCre) and inducible Cre‐ERT/2 and Cre‐ER lines driven by the same promoter were differentiated by selecting primers that anneal within the human or mouse ER ligand‐binding domains. Each primer set designed and described below is specific for one Cre line (ie, it does not generate the correct‐size amplicon in the other lines described within this article). The sequence of each validated primer per Cre line, the reference for the study reporting it, and the size of each PCR amplicon are provided in Table 1.

### Cre lines targeting the mesenchymal lineage

#### Wnt1

**Figure 3:**
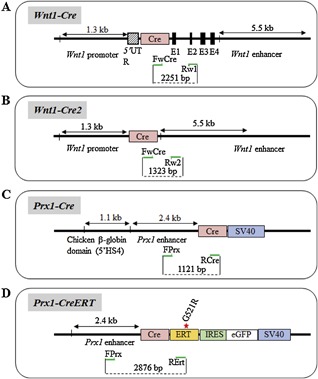
Cre lines based on the use of the *Wnt1* and *Prx1* promoters. (A‐D) Approximative structure and size of the promoter/enhancer and Cre sequences used for each construct. Primer location is indicated as green arrows.

Three different Cre‐transgenic lines have been developed based on the use of the *Wnt1* promoter to interrogate the role of genes involved in the formation and migration of neural crest cells, as well as midbrain and craniofacial development.^23^ Two lines have been generated by inserting using a knock‐in strategy either the Cre (*Wnt1‐Cre*; Fig. 3 *A*) or the Cre‐ER^TM^ (*Wnt1‐CreER*) transgenes before exon 1 of the *Wnt1* gene.^22^ The third (*Wnt1‐Cre2*) line was generated by inserting the Cre transgene between the 1.3‐kb promoter and the 5.5‐kb *Wnt1‐*enhancer sequence (Fig. 3 *B*).^24^ To distinguish between the *Wnt1‐Cre* lines from the *Wnt1‐Cre2* line, a common forward primer (FwCre) for both constructs was designed at the 3’ extremity of the Cre sequence, and a reverse primer was selected in exon 2 of the *Wnt1* gene (Rw1) to allow amplification of the *Wnt1‐Cre* line (2251‐bp product, Fig.  1, lane A), but not the *Wnt1‐Cre2* (because of the absence of exon 2). A reverse primer designed at the 5’ extremity of the 5.5‐kb *Wnt1‐*enhancer sequence (Rw2) allows amplification of a 1323‐bp product for the *Wnt1‐Cre2* line, but not for the *Wnt1‐Cre* line because of the distant location of the corresponding primer sequence (>5000‐bp amplicon size) in the *Wnt1‐Cre* transgene that prevents amplification (Fig.  1 lane B and Table 1).

#### Prrx1

There are three Cre lines built on the 2.4‐kb *Prx1‐*enhancer sequence, allowing the expression of noninducible (2.4‐kb *Prx1‐Cre;* Fig. 3 *C*) or inducible Cre transgenes (*Prx1‐CreERT*; Fig. 3 *D* and *Prx1‐CreERT2*) in limb osteochondroprogenitors.^25^, ^26^, ^27^, ^28^ For the *Prx1‐Cre* line, a forward primer at the 5'extremity of the 2.4‐kb enhancer element of the *Prx1* gene (FPrx) and a reverse one at the 5’ extremity of the Cre sequence (RCre) were chosen, leading to an amplicon of 1121 bp. This primer set also amplifies DNA from the 2.4‐kb *Prx1‐CreERT* mice because they share the same promoter sequence. However, PCR results revealed that the *Prx1‐CreERT* amplicon was larger than the one from the noninducible *Prx1‐Cre* mice (about 200 bp), suggesting the presence of additional nonreported DNA sequences in the *Prx1‐CreERT* line (Fig.  1, lane D). To differentiate these two lines, a different reverse primer was thus chosen in the hERT moiety (RErt; Fig. 3 *D*) of the *Prx1‐CreERT* construct, leading to an amplicon of 2876 bp when coupled to FPrx at the 3’ extremity of the 2.4‐kb *Prx1‐*enhancer sequence (Fig.  1, lane E).

#### Sox9

**Figure 4:**
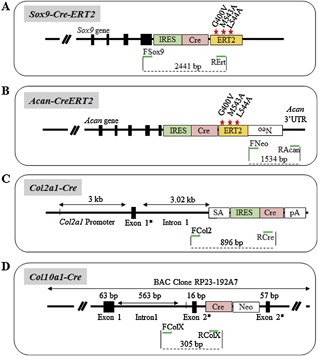
Cre lines based on the use of the *Sox9, Acan, Col2*, and *Col10* promoters. (A‐D) Approximative structure and size of the promoter/enhancer and Cre sequences used for each construct. Primer location is indicated as green arrows.

The inducible *Sox9‐CreERT2* mouse line was generated by inserting a *CreERT2* construct into the 3′ untranslated region (UTR) of the mouse *Sox9* gene (Fig. 4 *A*).^29^ To distinguish this transgenic mouse line from others, we designed a specific forward primer in exon 3 of the *Sox9* gene (Fsox9) and a reverse primer located in the hERT2 moiety (RErt). PCR amplification produces a 2441‐bp fragment (Fig.  1, lane *C*; Table 1).

#### Acan

There is, to our knowledge, only one aggrecan promoter‐driven line generated to express the Cre recombinase in chondrocytes.^30^ This line is a knock‐in of Cre into the 3'UTR of the *Aggrecan* gene (Fig. 4 *B*). To distinguish this transgenic line from others, a forward primer was chosen at the 3’ extremity of the neomycin cassette used for the construction of the knock‐in (FNeo) and was coupled to a reverse primer (RAcan) annealing at the 5’ extremity of the *Acan* 3'UTR, generating a fragment of 1534 bp (Fig.  1, lane *F*; Table 1).

#### Col2a1

Five different Cre transgenic lines have been generated using the *Col2a1* gene promoter /enhancer elements to control Cre recombinase expression in cartilaginous tissues. Three of them are constitutive,^31^, ^32^, ^33^ and the *Col2a1*‐CreERT^34^ and *Col2a1*‐CreERT2 35 lines are inducible via tamoxifen injection. Specific primers for the *Col2a1*‐*Cre* constitutive line generated in the Berhinger laboratory^31^ (Fig. 4 *C*) were designed with a forward primer annealing in the 1rst intron of the *Col2a1* gene (FCol2) and a specific reverse at the 5'extremity of the Cre recombinase sequence (RCre), producing a PCR amplicon of 896 bp (Fig.  1, lane *G*; Table 1).

#### Col10a1

Four different Cre transgenic lines have been generated using the *Col10a1* gene promoter to control Cre‐recombinase expression in hypertrophic chondrocytes,^36^, ^37^, ^38^ of which one is inducible.^39^ Specific primers for the *Col10a1*‐*Cre* line^38^ (Fig. 4 *D*) were designed with a forward primer annealing in the first intron of the *Col10a1* promoter (FColX) and a reverse primer at the 5’ extremity of the Cre recombinase sequence (RColX), creating a PCR product of 305 bp (Fig.  1, lane *H*; Table 1). Using these primers with the common PCR conditions to all primer sets validated in this report, a band was also generated with the gDNA of the *hCD11b‐Cre* transgenic mouse line. Reducing elongation time eliminated this band with the *hCD11b‐Cre* gDNA, but not with the *Col10a1* gDNA, thus demonstrating its nonspecific nature (Supplementary Fig.  1).

#### Osx

**Figure 5:**
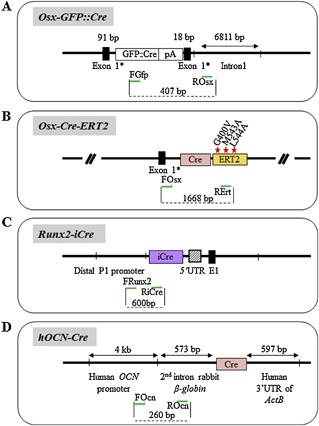
Cre lines based on the use of the *Osx, Runx2*, and *hOcn* promoters. (A‐D) Approximative structure and size of the promoter/enhancer and Cre sequences used for each construct. Primer location is indicated as green arrows.

The *Osterix* promoter was used to express the Cre recombinase in osteoprogenitors in the *Osx‐GFP*::*Cre* (Fig. 5 *A*) and the *Osx‐CreERT2* (Fig. 5 *B*) lines.^40^, ^41^ Primers specific for the *Osx‐GFP*::*Cre* line were reported in the original study and amplify a product of 407 bp (Fig.  1, lane N; Table 1). This primer set (FGfp and ROsx) was tested against the genomic DNA from our available DNA samples and was confirmed to be specific for the *Osx‐GFP*::*Cre* line. A forward primer in the BAC: RP23‐399N14 sequence (FOsx) was selected with a reverse primer specific to the ERT2 moiety (RErt) to specifically amplify an amplicon of 1668 bp in the *Osx‐CreERT2* line (Fig.  1, lane O; Table 1).

#### Runx2

One *Runx2‐Cre* mouse line is available to target genes in osteochondroprogenitors.^42^ The Cre recombinase under control of the *Runx2* promoter (BAC clone 150 kb) is the modified version of Cre (iCre),^43^ which has so far been less used in the bone field than the “regular” cre (although it has been used for generating Cre‐driver lines to investigate axial skeleton development^44^, ^45^). We chose a forward primer in the distal P1 promoter of *Runx2* (FRunx2) and a reverse primer at the 5’ extremity of the iCre sequence (RiCre; Fig. 5 *C*), yielding an amplicon of 600 bp (Fig.  1, lane 1; Table 1). Note that the generic Cre‐recombinase primer set does not amplify iCre (Fig.  1, *2).

#### Ocn

The *hOCN‐Cre* transgenic mouse was generated by cloning a 4‐kb fragment of the human *Osteocalcin* promoter upstream of the Cre recombinase sequence (Fig. 5 *D*).^46^ Primers designed to genotype this transgenic mouse line are described in the original article. The forward primer (FOcn) is positioned in the human *Osteocalcin* promoter sequence and the reverse primer is in the rabbit *B‐globin* intron sequence inserted between the promoter and the Cre‐recombinase sequence (ROcn). This primer set results in a PCR product of 260 bp and specificity of amplification was confirmed against other Cre lines available (Fig.  1, lane J; Table 1).

#### Col1a1

**Figure 6:**
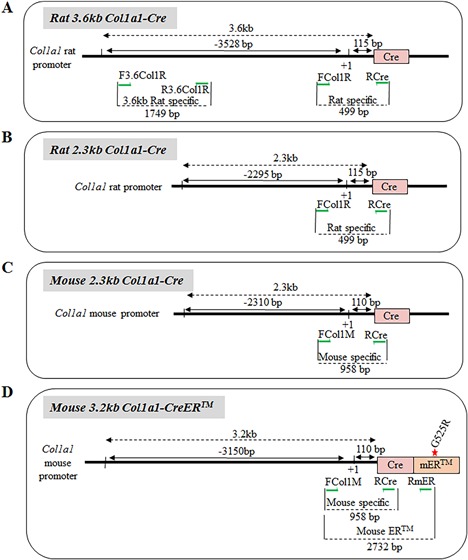
Cre lines based on the use of the *Col1a1* promoter. (A‐D) Approximative structure and size of the promoter/enhancer and Cre sequences used for each construct. Primer location is indicated as green arrows.

The *Col1a1* promoter was used to drive Cre expression in committed osteoblasts in four different mouse lines. Two are derived from the mouse *Col1a1* promoter,^41^, ^47^ and two from the rat *Col1a1* promoter.^48^ Promoter constructs of different size (2.3 kb, 3.2 kb, and 3.6 kb) have been used, and one of the mouse lines is inducible with the ER moiety (Fig. 6 *A*–*D*). Therefore, several independent strategies were required to establish specific primer sets for each of these *Col1a1*‐*Cre* lines.

### Rat versus mouse *Col1a1‐cre*

Our approach was first to verify the species origin of each *Col1a1‐Cre‐*line DNA made available to us. Because the mouse and rat promoters have 98% sequence homology, we selected forward primers in rare nonhomologous regions and used a common reverse primer in the 5’ Cre sequence (RCre). Two forward primers were designed (Fig. 6): one specific to the rat promoter sequence (FCol1R, 499‐bp amplicon), the other to the mouse promoter sequence (FCol1M, 958‐bp amplicon). Both primer pairs can distinguish rat and mouse species and are specific to the *Col1a1* lines (Fig.  9*A*, gel *A*, lanes 1 to 2; and gel *B*, lanes 3 to 4; Fig.  9*C*; Table 2).

### Rat 3.6‐kb‐*Col1a1‐Cre* versus rat 2.3‐kb‐*Col1a1‐cre*

Promoter fragments of different lengths were used by Kream and colleagues to generate these rat *Col1a1* transgenic mice.^48^ The classical design strategy, based on the selection of a forward primer in the *Col1a1* promoter and a reverse primer in the Cre transgene, was used to amplify the rat 2.3‐kb‐*Col1a1‐Cre* sequence, giving rise to a 499‐bp amplicon (FCol1R and RCre). This primer set, because of its proximal location in the *Col1a1* promoter, also amplifies the rat 3.6‐kb‐*Col1a1* line. Therefore, to discriminate the 3.6‐kb‐*Col1a1* mouse line, we took advantage of the large size of the rat 3.6‐kb‐*Col1a1* promoter to design a pair of primers with the forward primer sequence located in the distal 5’ region of the rat 3.6‐kb‐*Col1a1* promoter (F3.6Col1R) and a reverse primer located at the 5’ of the 2.3‐kb fragment (R3.6Col1R; Fig. 6 *A*). This primer pair gives rise to a PCR product of 1749 bp (Fig.  9*A*, gel C, lane 1; Table 2).

### Mouse 3.2‐kb *Col1a1 Cre‐ER* and mouse 2.3‐kb *Col1a1 Cre*

To differentiate between these two transgenic lines, we selected a common forward primer located in the proximal region of the mouse *Col1a1* promoter (FCol1M) coupled with reverse primers located in either the 5’ Cre sequence (RCre, mouse 2.3‐kb *Col1a1‐Cre*) or the ligand‐binding domain of the mouse estrogen receptor (RmER, mouse 3.2‐kb *Col1a1 Cre‐ER^TM^* Fig. *6C,D*). This creates 958‐bp and 2732‐bp‐long PCR products, respectively (Fig.  9*A*, gel B, lane 3 and gel D, lane 4; Table 2).

#### Dmp1

**Figure 7:**
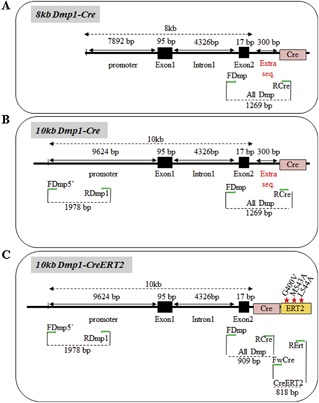
Cre lines based on the use of the *Dmp1* promoter. (A‐C) Approximative structure and size of the promoter/enhancer and Cre sequences used for each construct. Primer location is indicated as green arrows.

There are three Cre lines built on the use of the mouse *Dmp1* promoter, generated to drive Cre expression in osteocytes. Two use a so‐called 10‐kb promoter, coupled with either the regular Cre sequence or with Cre‐ERT2.^49^, ^50^ Of note, these 10‐kb *Dmp1*‐*Cre* mouse lines are sometimes called the 15‐kb Dmp1‐Cre lines because of the use of the 10‐kb promoter plus a 5‐kb element‐containing exon 1 (95 bp), intron 1 (4326 bp) and the noncoding region of exon 2 (17 bp).^49^ The other *Dmp1* line utilizes the so‐called 8‐kb promoter (actually rather 12 kb) coupled to the regular Cre sequence (Fig. 7 *A–C*).^51^, ^52^

The design of primer sets to discriminate these lines from each other required a stepwise strategy. First, we designed a primer set that was able to recognize the *Dmp1* promoter. We achieved this by choosing a common forward primer with its sequence located in intron 1 (FDmp) coupled with a reverse primer placed in the common 5’ Cre sequence (RCre), leading to a calculated amplicon of 909 bp. Unexpectedly, different amplicon sizes were obtained between *Dmp1‐Cre* lines (909 bp for the 10‐kb *Dmp1‐CreERT2* line and ∼1260 bp for the 8‐kb and 10‐kb *Dmp1‐Cre* lines, despite the fact that all cloned elements controlling the Cre recombinase were supposedly identical between the three transgenic mice (Fig.  9*B*, gel A; Table 3). After purification and sequencing of these amplicons, it was concluded that the 8‐kb and the 10‐kb *Dmp1‐Cre* constructs had a supplemental sequence (matching with the sequence of the cloning vector Flp recombinase; Genbank: U46493.1) of approximately 300 bp upstream of the 5’ extremity of the Cre recombinase that was not included in the 10‐kb *Dmp1‐Cre ERT2* line (see Fig. 7). Nevertheless, this primer set is specific to these *Dmp1‐Cre* transgenic lines (Fig.  9*C*).

We then designed a primer set to dissociate the 10‐kb versus the 8‐kb *Dmp1‐Cre* lines. A forward primer at the 5’ extremity of the 10‐kb *Dmp1* promoter (FDmp5’) and a reverse primer common to the 10‐kb and 8‐kb *Dmp1‐Cre* promoter constructs (RDmp1) were selected and produce, an amplicon of 1978 bp specific to the 10‐kb *Dmp1‐Cre* lines (Fig. 7 and Fig. 9 *B*, gel B; Table 3). Unfortunately, this primer set amplified the 8‐kb *Dmp1‐Cre* DNA as well, suggesting that the related construct shared this region of the promoter with the 10‐kb *Dmp1‐Cre* construct.

To discriminate between the 10‐kb *Dmp1‐Cre* and the 10‐kb *Dmp1‐CreERT2* lines, a common forward primer (FwCre) was designed at the 3’ extremity of the Cre sequence, and a specific reverse primer was selected in the Cre‐ERT2 moiety (RErt). The use of these primers yields no amplification with the noninducible 10‐kb *Dmp1‐Cre* transgene, but does amplify an 818‐bp fragment with DNA from the 10‐kb *Dmp1‐CreERT2* transgene (Fig.  9*B*, gel C, lane 3; Table 3).

### Cre lines targeting the hematopoietic lineage

#### CD11b

**Figure 8:**
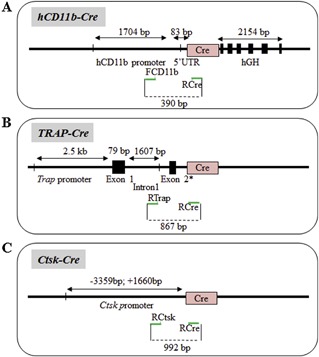
Cre lines based on the use of the *hCD11b, TRA*, and *Ctsk* promoters. (A‐C) Approximative structure and size of the promoter/enhancer and Cre sequences used for each construct. Primer location is indicated as green arrows.

The *hCD11b‐Cre* transgenic mouse was generated by cloning the 1.7‐kb fragment of the human *CD11b* promoter upstream of the Cre recombinase sequence,^53^ allowing the expression of the Cre recombinase in the myeloid‐osteoclast lineage (Fig. 8 *A*). We designed a specific forward primer in the human *CD11b* promoter (FCD11b) and a reverse primer located at the 5’ extremity of the Cre sequence (RCre) to amplify a 390‐bp PCR product (Fig. 1, lane *K*; Table 1).

#### TRAP and cathepsin K

To evaluate the involvement of genes in mature osteoclasts specifically, two transgenic mouse models have been generated: the tartrate‐resistant acid phosphatase (*Trap*; Fig. 8 *B*) and the cathepsin‐K (*CtsK*; Fig. 8 *C*) Cre lines.^54^ Primers allowing the distinction of these two transgenic mice have been previously described (although an inversion between the forward primers for the FTrap and FCtsk promoters was noted in the original article 54). The primer sets for the *Trap‐Cre* and *Ctsk‐Cre* transgenic mice produce a fragment of 867 bp and 992 bp, respectively (Fig. 1, lanes L and M, respectively; Table 1).

**Figure 9:**
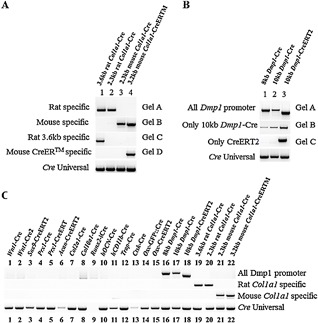
PCR amplicons used to discriminate the *Col1a1* and *Dmp1* promoter constructs. (*A*) Primers set PCR products to discriminate the different *Col1a1*‐Cre transgenic mice. (*B*) Primers set PCR products to discriminate the *Dmp1*‐Cre transgenic mice. (*C*) Validation of the specificity of *Col1a1* and *Dmp1* primers against other available Cre transgenic lines.

### HRM PCR to discriminate the ERT or ERT2 transgenes

**Figure 10:**
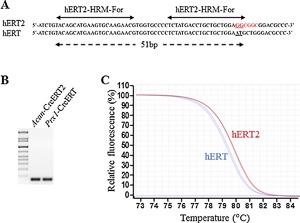
High‐resolution melting curves analysis to dissociate the Cre‐ERT and Cre‐ERT2 transgenes. (*A*) Primers and product (51 bp) amplified from the hERT and hERT2 transgenes (red codons correspond to the mutation for M543A and L544A, respectively). (*B*) Identical migration profile of the PCR products amplified from the hERT2‐HRM reaction with *Acan‐CreERT2* and *Prx1‐CreERT* purified gDNAs (51 bp). (*C*) Fluorescence normalized high‐resolution melting curves from the hERT2 (red) and hERT (blue) HRM amplifications from *Acan‐CreERT2* and *Prx1‐CreERT* gDNAs (*n* = 2 per genotype).

The *Col2* and *Prx*‐inducible Cre lines exist in two forms, each with either an ERT or ERT2 moiety. The high‐sequence similarity and GC content of these moieties did not allow us to successfully discriminate the presence of ERT versus ERT2 in these two lines. We therefore used an HRM PCR approach to achieve this goal. Primers selected to discriminate the ERT and ERT2 transgene are illustrated in Fig. 10 *A*. Two different genomic DNAs from *Acan‐CreERT2* and *Prx1‐CreERT* mouse tails were used to validate the hERT2‐HRM primers. Although conventional PCR with these primers generated, as expected, a similar‐sized amplicon (51 bp; Fig. 10 *B*) and owing to the fact that the hERT2 transgene possesses a higher melting temperature because of its higher GC content (GGC versus ATG), fluorescence normalized HRM curves plots clearly discriminated between the two different transgenes (Fig. 10 *C*).

## Discussion

We have successfully designed primer sets specific for 15 Cre‐deleter mouse lines commonly used in the bone field, as well as additional primer sets able to discriminate lines built on similar promoters and/or Cre sequences. This approach can be used alongside, or instead of, generic Cre‐recombinase primer sets to authenticate specific mouse lines and avoid accidental swapping of mouse lines within or between laboratories.

The goal of this work was not to validate and provide an exhaustive list of conditions and validated primer sets, as Cre transgenic lines are continuously being created. Our goal was rather (1) to raise awareness of the potential issue of using a generic Cre primer set; (2) to demonstrate the feasibility of generating specific primers sets for each Cre line; and (3) to incite investigators generating new Cre lines to design such primer sets or appropriately report the details of the constructs generated to allow this design by others. Lacking from this list are a number of Cre lines targeting chondrocytes (*Col2a1‐cre*), skeletal stem cells (*Nestin‐cre, ObRb‐cre, Mx1‐cre, Gremlin‐CreERT, Wnt1‐CreER*), differentiated osteoblasts (*hOCN*‐*CreERT2*), or tendinocytes (*Scleraxis‐Cre*).^31^, ^32^, ^35^, ^55^, ^56^ PCR‐primer‐sets validation is not expected to be challenging for most of them. For instance, different primers for the two other constitutive *Col2a1*‐*Cre* lines not tested in this study can be designed based on their construction specificities. For example, a forward primer annealing at the 3’ extremity of the Cre sequence and a reverse primer chosen in exon 52 should be able to selectively amplify the Cre‐transgenic mouse created by Aszodi and colleagues^32^ based on the unique proximity between Cre and exon 52 specific to this *Col2a1*‐*Cre* construct. The *Col2a1*‐*Cre* line created in the St‐Arnaud laboratory has the Cre‐recombinase transgene inserted between the 3‐kb‐*Col2a1* promoter and the 3‐kb‐enhancer elements of the *Col2a1* gene, and thus should not be amplified by the primers used for genotyping the *Col2a1‐Cre* mice from Aszodi's^32^ and Berhinger's^31^ laboratories. The two inducible *Col2a1*‐*Cre* lines use common regulating elements to control the expression of the Cre‐ERT/2 cassette. However, in the *Col2a1*‐*Cre‐ERT* construct, a beta‐globin sequence has been inserted between the 1‐kb *Col2a1* promoter and the 5’ extremity Cre sequence, which is not present in the *Col2a1*‐*Cre*‐*ERT2*. This characteristic can be used to design a specific set of primers recognizing only the *Col2a1*‐*CreERT* line. Because the *Wnt1‐Cre* and *Wnt1‐Cre‐ER^TM^* constructs have the same regulatory elements,^22^ a forward primer specific for the mouse estrogen receptor coupled to the reverse primer annealing in exon 2 can be used to generate a primer set specific for the Cre‐ER^TM^ line.

More difficult was the generation of a primer set able to discriminate CreERT and CreERT2 lines that share the same promoter (such as the *Prx1* and *Col2a1*‐*Cre* lines). These two CreERT sequences are characterized by a high‐sequence similarity (only two nucleotides, GGC à CGG, are mutated in the G521R construct) and the sequence surrounding the double‐mutation M543A‐L544A (ATGCTG à GCGGCG) in the Cre‐ERT2 construct is characterized by high (>80%) GC content. A HRM curve PCR was the only strategy able to successfully discriminate these two transgenes.^57^

In an effort to simplify genotyping protocols, PCR amplifications were all performed with the same conditions (master mix used, annealing temperature, elongation time, number of cycles). Because amplicon size and optimal annealing temperature differ between primer sets, this unique protocol generated in some cases additional bands whose nonspecific nature could easily be determined based on incorrect size and lower intensity. The *Col10a1* primer set was the only exception, as it generated a band at the proper size with the gDNA from the *hCD11b‐Cre* mice. However, this band disappeared by reducing PCR elongation time.

A recurring difficulty encountered during this work was to find out the exact genomic structure of the constructs used to generate Cre transgenic lines. In multiple cases, a very vague description of the strategies and sequences used was available, making the design of specific primer sets a challenging and time‐consuming endeavor. This prevented us from successfully validating primer sets able to discriminate the 8‐kb and 10‐kb *Dmp1‐Cre* lines. It is therefore worth emphasizing the need for a precise description of generated constructs and Cre lines and for the sharing of related complete sequences when reporting a new transgenic line (in addition to more efforts in characterizing the possible effects of Cre genomic insertion and Cre “leakage” in nontargeted tissues).

The use of the Cre‐lox approach to generate genetic conditional KO mouse models allowed exquisite control of gene expression that has led scientists to unravel numerous new biological pathways. This approach is not without caveats: The proper authentication of Cre‐deleter lines, recognition of the limitations of this approach, and the use of additional and independent strategies to assess gene function, including pharmacological and ex vivo approaches, are all critical for scientific rigor and the appropriate interpretation of experimental results.

## Disclosures

The authors have no conflict of interest to disclose.

## Supporting information

Additional supporting information may be found in the online version of this article at the publisher's web‐site

Supporting Figure S1.
