# Supplementary figures and images for: Promoter Cre‐Specific Genotyping Assays for Authentication of Cre‐Driver Mouse Lines
Source: JBMR Plus. 2019 Jan 18;3(4):e10128. doi: 10.1002/jbm4.10128 (PMC6478581; doi:10.1002/jbm4.10128)

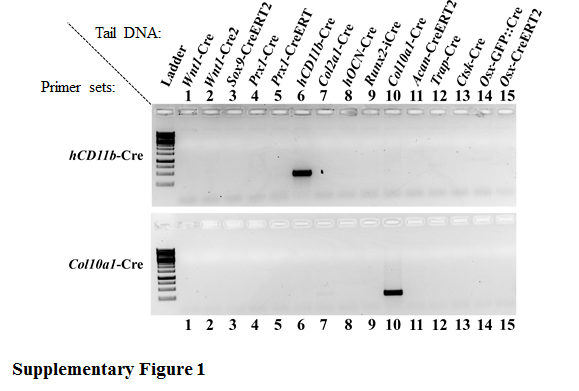

Supplement: Supplementary file 1 — Supporting Figure S1. [file JBM4-3-na-s001.tif]
